# Effects of Air Pollution on Dialysis and Kidney Transplantation: Clinical and Public Health Action

**DOI:** 10.3390/jcm14207194

**Published:** 2025-10-12

**Authors:** Sławomir Jerzy Małyszko, Adam Gryko, Jolanta Małyszko, Dominika Musiałowska, Anna Fabiańska, Łukasz Kuźma

**Affiliations:** 1 Department of Physiology and Pathophysiology, Medical University of Warsaw, 02-091 Warsaw, Poland; smalyszko1999@gmail.com; 2 Department of Invasive Cardiology, Medical University of Bialystok, M. Sklodowskiej-Curie 24a, 15-276 Bialystok, Poland; adamboleslaw@gmail.com (A.G.); kuzma.lukasz@gmail.com (Ł.K.); 3 Department of Nephrology, Dialysis and Internal Medicine, Medical University of Warsaw, 02-097 Warsaw, Poland; anna.fabianska@wum.edu.pl; 4 Department of Cardiology, Lipidology and Internal Diseases, Medical University of Bialystok, Żurawia 14, 15-569 Bialystok, Poland; dominika.musialowska@umb.edu.pl; 5 Department of Cardiac Surgery and Transplantology, National Medical Institute of the Ministry of Interior, 02-507 Warsaw, Poland; 6 Liverpool Centre for Cardiovascular Science, University of Liverpool, Liverpool John Moores University and Liverpool Heart & Chest Hospital, Liverpool L14 3PE, UK

**Keywords:** air pollution, hemodialysis, peritoneal dialysis, kidney transplantation, exposure

## Abstract

Air pollution is associated with many adverse health outcomes, including kidney diseases. Kidney diseases, especially chronic kidney disease, are a significant public health issue globally. The burden of kidney disease is expected to rise due to population aging and the growing prevalence of diabetes and hypertension. End-stage kidney disease is associated with significant healthcare costs, morbidity, and mortality. Long-term exposure to air pollution was associated with increased risk for chronic kidney disease progression to kidney replacement therapy. Evidence on the effect of short-term exposure to air pollution on renal function is rather limited. Kidney transplant patients are likely to be even more susceptible to detrimental effects of air pollutants. Exposure to air pollution results in a higher risk for delayed graft function, acute rejection, and mortality. In this review we would like to summarize the state of knowledge on the influence of air pollution on outcomes in end-stage kidney failure and kidney transplantation.

## 1. Introduction

Air pollution is associated with many adverse health outcomes, including kidney diseases [1,2,3,4,5,6,7,8,9,10,11,12]. Cohen et al. [13] explored spatial and temporal trends in mortality and burden of disease attributable to ambient air pollution from 1990–2015 at global, regional, and country levels. In their analysis of data from the Global Burden of Disease Study 2015 they showed that ambient air pollution contributed to nearly 8% of all deaths, and this phenomenon increased with time—predominantly due to increases in fine particulate matter (PM) and mortality from non-communicable diseases (NCDs), mainly in large low-income and middle-income countries (LMICs) due to growth and aging of their populations [13]. The World Health Organization (WHO) updated air quality guidelines in 2022 [14,15]. The European Environment Agency in 2021 recorded 239,000 premature deaths due to chronic exposure to fine particulate matter (i.e., exposure to PM_2.5_ levels over the WHO guideline of 5 µg/m^3^), 48,000 because of chronic nitrogen dioxide exposure (i.e., O_3_ levels over the WHO guideline of 60 µg/m^3^ and exposure to NO_2_ levels over the WHO guideline of 10 µg/m^3^), and 70,000 due to short-term ozone exposure (i.e., O_3_ levels over the WHO guideline of 60 µg/m^3^ and exposure to NO_2_ levels over the WHO guideline of 10 µg/m^3^), respectively [15,16]. The relative risks were derived from epidemiological studies for populations above a certain age (more than 30 years old for PM_2.5_ and NO_2_ and more than 25 years old for O_3_ levels). The European Environment Agency (EEA) also estimated the number of attributable deaths and the attributable deaths per 100,000 inhabitants at risk in the year of 2021. Sensitivity analysis of attributable deaths was performed by the agency considering the full range of exposures to PM_2.5_ and NO_2_, i.e., all levels above 0 µg/m^3^. Additionally, in a secondary sensitivity analysis, the agency estimated deaths attributable to annual exposure to PM_2.5_ and NO_2_ levels over 10 µg/m^3^ and 20 µg/m^3,^ respectively [17]. As there is no EU legal standard linked to exposure to O_3_ in peak seasons, ozone exposure was not assessed in the sensitivity analysis [17].

Updated estimates from the State of Global Air 2024 (based on GBD 2021) indicate that air pollution was the second-leading risk factor for death worldwide in 2021, accounting for approximately 8.1 million deaths. Notably, substantial health burdens persist even at concentrations below the 2021 WHO guideline values, with the largest absolute impacts observed in LMICs and PM_2.5_ remaining the dominant contributor [18].

Air pollution comes from a complex mixture of both gaseous and solid components together with liquid particles in the atmosphere as a primary result of coal, gasoline, and diesel fuel combustion [19]. The main components of atmospheric pollution are gaseous compounds, nitrogen dioxide, carbon monoxide (from road traffic and the burning of industrial fuels), ozone (O_3_), and sulfur oxides (from industries) [20]. Based on the particles’ nature, PM can be classified as biological, chemical, mineral, or metallic. Air pollutants are divided in relation to the aerodynamic size as thoracic particles (≤10 µm)—PM_10_, fine fraction (≤2.5 µm)—PM_2.5_, coarse fraction (10–2.5 µm)—PM_10–2.5_, and ultrafine particles (<0.1 µm)—UFP [21]. In addition, other air pollutants, such as environmental tobacco smoke (ETS) and gaseous heavy metals, appear to be responsible for kidney injury [21]. These gases alter gene expression in inflammatory signalling, antioxidant response pathways, and endothelial dysfunction in kidneys. In addition, they also reduce blood flow in the kidneys and contribute to the rise in oxidative stress and inflammation, which can in turn damage DNA, leading to further injury [22,23]. Polluted air—particularly PM_2.5_ and NO_2_—affects the kidneys both directly and indirectly by amplifying established risk factors [24]. Direct effects: well-described pathophysiological mechanisms—including oxidative stress, endothelial dysfunction, renin–angiotensin–aldosterone system (RAAS) activation, inflammation, and tubulointerstitial fibrosis—translate into eGFR decline and albuminuria [24]. Recent syntheses and a meta-analysis of kidney function markers corroborate associations between long-term air-pollution exposure and deterioration of kidney function and CKD risk, with additional signals for AKI and KI-related mortality. Indirect effects: air pollution increases the risk and severity of hypertension and diabetes—the principal drivers of progression to KRT—as shown in large cohort studies and meta-analyses [25,26,27,28,29]. Chronic kidney disease (CKD) as defined by KDIGO [30] is well known as a cardiovascular disease risk factor, and its prevalence is on the rise due to an aging population and growing prevalence of diabetes and hypertension [31,32,33]. Over the last few years, growing attention has been paid to the association between environmental exposures and CKD and its progression [25,34,35,36], however, effects of air pollution in ESKD patients undergoing dialysis or kidney transplantation are understudied. As the leading environmental risk factor, the global burden of disease attributed to air pollution is enormous. Polish smog is a specific type of air pollution present in eastern Poland, which may cause particularly adverse cardiovascular effects, and the ED-PARTICLE study showed a strong association between air pollution and cardiovascular disease and mortality as well as kidney diseases [37,38,39,40,41].

In this review, we will present the epidemiologic observations linking air pollutant exposure to the development and progression of CKD to ESKD. Then, we discuss the potential roles of various air pollutants, including particulate matter and gaseous co-pollutants, environmental tobacco smoke, and gaseous heavy metals, in its pathogenesis. Finally, this review outlines the latent effect of air pollution in ESKD patients undergoing dialysis or renal transplant.

## 2. The Role of Air Pollutants in ESKD Patients

Feng et al. [42] assessed the relation between air pollution (expressed as annual PM_2.5_ level at the ZIP code level) and all-cause mortality. They finally studied 384,276 older patients who initiated dialysis from 2010–2016. They linked elderly patients with kidney failure using the US Renal Data System and PM_2.5_ data (obtained from NASA’s Socioeconomic Data and Application Center (SEDAC) Global Annual PM_2.5_ Grids from NASA Moderate Resolution Imaging Spectroradiometer, Multi-angle Imaging Spectroradiometer, and the Sea-Viewing Wide Field-of-View Sensor Aerosol Optical Depth with Geographically Weighted Regression) through the year of first dialysis and their ZIP code of residence. They found that, in dialysis patients, exposure to PM_2.5_ greater than 12 μg/m^3^ was associated with increased mortality in older adults [42]. Even if the PM_2.5_ level was below the national ambient air quality standard, the risk was increased for the elderly >75 years old [42]. They also reported that, in elderly female or Black patients or patients with diabetic nephropathy as a cause of ESKD, PM_2.5_ was associated with a higher mortality risk [42]. According to Xi et al. [43], wildfires were also increasingly a major source of PM_2.5_. An exposure to PM_2.5_ was positively associated with all-cause mortality [44]. Carpal tunnel syndrome, a known complication of dialysis treatment, was also positively associated with long-term PM_2.5_ exposure [44]. Another complication of dialysis, pruritus, was also positively linked to environmental NO_2_/CO exposure, for example, in a multivariate logistic regression, environmental NO_2_/CO exposure was positively linked to uremic pruritus [45]. In hemodialysis patients, exposure to PM_10_ was positively associated with brachial–ankle PWV [46], an independent risk factor for cardiovascular mortality in dialysis patients [47]. Similar data were published for peritoneal dialysis patients [48]. Huang et al. [48] assessed, in a single-center observational retrospective study on 175 PD patients, the relation between PD infections such as PD peritonitis (n = 18), tunnel infection, or exit site infection (n = 17) and environmental PM_2.5_ exposure. The most common pathogens in peritonitis were Streptococcus and Escherichia, whereas, for other types of infections, it was Staphylococcus. It is of interest that the environmental CO level was positively correlated with hs-CRP level in peritoneally dialyzed patients [48]. Moreover, high environmental exposure to PM_2.5_ resulted in a higher infection rate than in those with low exposure, underlining the fact that air pollution may be associated with peritoneal-dialysis-associated infection.

On the other hand, high exposure to PM_2.5_ was associated with acute pulmonary edema in non-dialytic patients with ESKD [47]. Chiu et al. [49] found this relation in 317 patients with stage 5 CKD hospitalized for acute lung edema (diagnosed based on respiratory distress with clinical signs of acute pulmonary edema in imaging studies and biochemistry including cardiac enzyme and natriuretic peptide levels) when the dialysis therapy was also initiated. They excluded patients with AKI, current myocardial infarction, decompensated heart failure, combined pneumonia, preexisting interstitial lung disease, and lung cancer. Interestingly, they also found that hot ambient temperatures in summer and low ambient temperatures in winter were also risk factors for acute pulmonary edema in this population of non-dialysis CKD stage 5 patients. In addition, CRP could serve as a marker for all-cause and cardiovascular mortality in hemodialyzed patients [50].

Hamroun et al. [51] in the French REIN registry nationwide cohort study included 90,373 adult kidney failure patients initiating maintenance dialysis between 2012 and 2020 and investigated the association of multiple exposures to air pollutants PM_2.5_, PM_10_, and NO_2_ with all-cause and cause-specific death in dialysis patients. They found that long-term multiple air pollutant exposure was associated with all-cause and cause-specific mortality in the dialysis population. Cesaroni et al. [28] also performed a registry-based study on the association of long-term exposure to nitrogen dioxide (NO_2_), fine particulate matter (PM_2.5_), black carbon (BC), and ozone (O_3_) with end-stage kidney disease incidence in two large population-based European cohorts, i.e., the Austrian Vorarlberg Health Monitoring and Promotion Program (VHM&PP, 136,823 individuals) and the Italian Rome Longitudinal Study (RoLS 1,939,461 individuals). Interestingly, in the Austrian cohort no evidence of an association between PM_2.5_ or O_3_ and end-stage kidney disease was observed, whereas in the Italian cohort PM_2.5_ exposure was associated with incidence of end-stage kidney disease. The absence of association in one cohort and presence in another may be due to different NO_2_ exposures levels, i.e., approximately 10 µg/m^3^ lower in Austria compared to Italy. Similar findings were reported for black carbon. Differences were smaller for PM_2.5_ and especially O_3_.

Kadelbach P et al. [52], in the European multicenter ELAPSE study on 289,564 persons followed for 20.4 years, found positive associations between exposure to PM_2.5_ and CKD-related mortality and the inverse for ozone. This study included only Western European cohorts with at least 10 cases of CKD-associated mortality (Diet, Cancer and Health cohort from Denmark (DCH), Danish Nurse Cohort (NC-1993), the prospective sub-cohort of the Dutch European Investigation into Cancer and Nutrition (EPIC_NL-Prospect), Etude Epidémiologique auprès de femmes de la Mutuelle Générale de l‘Education Nationale (E3N) from France, and Vorarlberg Health Monitoring and Prevention Programme (VHM&PP) from Austria). However, after exclusion of the largest sub-cohort contributing 226 cases, from Austria, associations became null. Lin et al. [53] assessed whether PM_2.5_ exposure was associated with progression of chronic kidney disease (CKD) to KFRT in a prospective cohort study. Here, 6628 adult patients with CKD aged 20 to 90 were recruited from the Advanced CKD Program in Taiwan between 2003 and 2015. According to them, the adjusted HR for progression to KFRT was 1.19 (95% CI, 1.08–1.31) per 7.8 µg/m^3^ increase in PM_2.5_, an amount spanning the interquartile range. Moreover, there was evidence of a dose–response relationship (adjusted HRs of 1.16 (95% CI, 0.90–1.51), 1.19 (95% CI, 0.94–1.52), and 1.42 (95% CI, 1.12–1.80) for low, medium, and high PM_2.5_ levels), whereas no significant association between PM_2.5_ and all-cause mortality (adjusted HR, 1.01 (95% CI, 0.95–1.08)) was found. Couchoud et al. [54] studied the associations between high temperatures and mortality for patients with stage 5 chronic kidney disease on kidney replacement therapy either with dialysis or with a kidney transplantation in France using various definitions of elevated temperature. Between June and September, over the years 2012–2022, temperatures varied from 6.7 to 45.4 °C. During this period, 20,174 deaths were recorded among 116,808 dialysis patients and 3340 among 64,531 transplanted patients. The authors stressed that a maximum temperature >32.5 °C was associated with mortality and an incidence ratio (IR) of 1.09 (1.04–1.15) in the dialyzed population, whereas no such association was observed in kidney transplant recipients.

Data emerge on the role of prolonged heat exposure in the risk of chronic kidney disease of non-traditional etiology [55]. To date, in the published literature there is strong evidence that heat waves and high temperatures are critical risk factors for kidney-related morbidity and mortality [56,57,58,59,60]. A specific form of CKD, the Meso-American nephropathy epidemic, is a form of CKD of unknown origin that devastated agricultural workers, especially cutting sugarcane, in hot Pacific coastal regions along the coast of Central America [61,62,63]. This is one of the most alarming examples of heat-related kidney disease, where repeated AKI episodes evolved into CKD over time [61,62,63]. However, there is no data so far on the role of excessive heat exposure in kidney outcomes in kidney transplant recipients and in ESKD. The link with air pollutants is also not critically established and further research is needed to determine the exact role of heat stress in CKD of unknown origin and its possible progression to ESKD [64].

Recently, the term “exposome” was introduced to encompass a variety of factors, including personal behaviors like smoking, a sedentary lifestyle, and making specific dietary choices (i.e., consuming ultra-processed foods, etc), as well as pesticides, air, water, and soil pollution, nanoplastics, global warming, stressful life events, and socioeconomic status [65]. In a recent meta-analysis including 7,967,388 participants from both cohort and cross-sectional studies until 2023, Wathanavasin et al. [27] revealed that each 10 µg/m^3^ rise in airborne PM_2.5_ was associated with increased CKD incidence and prevalence. More importantly, exposure to PM_2.5_ was also related to the incidence of end-stage kidney disease, suggesting an increased risk when the duration of exposure lasted longer than 10 years [27]. In Table 1 other studies are presented. Showing duration of the study, population, study design, pollutants, and major findings.

## 3. Air Pollution and Kidney Transplantation

Despite the existing data on associations between air pollution, in particular, levels of PM_2.5_, and health outcomes, evidence of an association between air pollution, predominantly levels of PM_2.5_, and the outcomes of solid organ transplantation is very limited. Bhinder et al. [77] reported that increased levels of PM_2.5_ were associated with an increased risk of chronic lung allograft dysfunction and overall mortality. The same findings have been found among heart transplants recipients [78]. In kidney transplant recipients, exposure to air pollutants was associated with an increased risk of cardiovascular death, however, associations between PM_2.5_ levels and other important transplant outcomes have not been studied so far [79,80].

Spencer-Hwang et al. [79] performed a retrospective cohort study on 32,239 non-smoking adult kidney transplant recipients undergoing transplantation from 1997–2003. They were identified through the US Renal Data System and living in the United States within 50 km of an air pollution monitoring station. They assessed long-term ambient pollutant O_3_ and particulate matter ≤10 µm (PM_10_) using monthly concentrations of O_3_ and PM_10_ calculated from ambient monitoring data by the US Environmental Protection Agency Air Quality System and interpolated to ZIP code centroids according to patients’ residences. They found 267 cardiovascular deaths and 2076 deaths from natural causes during the 7-year study follow-up. They calculated risk of fatal chronic heart disease for each 10 ppb O_3_ increase. They found that this risk increased by 35% (RR, 1.35; 95% CI, 1.04–1.77) in the single-pollutant model and 34% (RR, 1.34; 95% CI, 1.03–1.76) in the two-pollutant model. On the other hand, there were no independent associations between chronic heart disease and PM_10_ as well as between levels of PM_10_ or O_3_ and natural-cause mortality (RR, 1.09; 95% CI, 0.99–1.21). In addition, Spencer-Hwang et al. [69] also stressed that females consistently experienced greater risk than males for fatal coronary heart disease upon exposure to O_3_ and PM_10_.

The most recent study by Han et al. [81], a multicenter, longitudinal cohort study, investigated the long-term O_3_ exposure effects on kidney transplantation outcomes by employing a multipollutant model. They included 4796 adult kidney transplant recipients undergoing kidney transplantation from 2002–2020 at three major hospitals in South Korea and surviving more than one year. They estimated exposures to O_3_ and PM_2.5_ using residential ZIP-code-based high-resolution machine learning models. They evaluated associations between exposure to O_3_ and all-cause mortality or death-censored graft failure using time-varying Cox proportional hazards models adjusted for potential confounders, including PM_2_._5_. They found that the mean annual levels of O_3_ and PM_2_._5_ one year after kidney transplantation were 38.9 ± 11.3 µg/m^3^ and 26.7 ± 6.8 µg/m^3^, respectively. They also reported that a 5 ppb increase in levels of O_3_ was related to enhanced risks of death-censored graft failure (hazard ratio (HR) = 1.60; 95% confidence interval (CI) = 1.40–1.82) and all-cause mortality (HR = 1.65; 95% CI = 1.36–2.00). More importantly, these associations remained consistent across sub-group and sensitivity analyses. The association of O_3_ with adverse outcomes in kidney transplant recipients underlies and extends our understanding of the broad health consequences of air pollution. These studies were focused on the possible associations between air pollution and mortality in kidney transplant recipients. However, data on the effects of air pollution on graft function, acute rejection or death-censored graft failure (DCGF), and all-cause mortality are very scarce.

Kim et al. [82] investigated potential associations between these data and the clinical outcomes of 1532 kidney transplant recipients undergoing transplantation in a tertiary hospital between 2001 and 2015. In this Korean study [82], air pollutant data were obtained from the Korean National Institute of Environmental Research. The authors found that an increase in annual PM_10_ concentration was significantly associated with higher risk of death-censored graft failure (for 1 mcg/m^3^ increase, HR = 1049), biopsy-proven rejection (HR = 1053), and all-cause mortality (HR = 1.09) [82]. In their fully adjusted model, they reported that all-cause mortality was significantly associated with 1-year average PM_10_ levels (HR, 1.09; 95% CI, 1.043 to 1.140). In a US study, Feng et al. [83] using data for 87,233 adult kidney transplant recipients from the Scientific Registry of Transplant Recipients (1 January 2010–31 December 2016), estimated annual ZIP-code-level PM_2.5_ concentrations at the time of transplantation using NASA’s SEDAC Global PM_2.5_ Grids. They found that PM_2.5_ was associated with higher odds of delayed graft function and 1-year acute rejection and elevated risk of mortality among kidney transplant recipients. indicating that environmental exposure to air pollutants could be regarded as a prognostic risk factor after engraftment. In addition, high exposure to ambient PM_2.5_ was also associated with cardiovascular disease and coronary heart disease death. It is of interest that these associations were more significant for black recipients relative to non-blacks for cardiovascular mortality [84]. Chang et al. [85] conducted a retrospective cohort study using data on patients who underwent kidney transplantation from 2004 to 2016 and were identified in the national US transplant registry and followed up through March 2021. Exposures included posttransplant time-dependent annual mean level of PM_2.5_ (in 10 µg/m^3^) and mean level of PM_2.5_ in the year before transplantation (i.e., baseline levels) in quartiles, as well as baseline annual mean level of PM_2.5_ (in 10 µg/m^3^). They showed that annual PM_2.5_ concentration was a risk factor for acute rejection, graft failure, and all-cause mortality in a cohort study of 112,098 patients with kidney transplants. Li et al. [86] studied association between PM_2.5_ and post-KT mortality by neighborhood racial and ethnic segregation in a population of 185,872 non-Hispanic Asian, non-Hispanic Black, Hispanic, and non-Hispanic White adult (18 years or older) KT recipients (2000–2021) from the Scientific Registry of Transplant Recipients (SRTR). They found that high exposure to PM_2.5_ was significantly associated with increased mortality among kidney transplant recipients in high-segregation neighborhoods. In a Korean study on 232 living kidney donors with 2 years of follow-up, peri-operative exposure to PM_2.5_ was associated with the deterioration of renal function in these subjects. In addition, a 1 µg/m^3^ increase in mean level of PM_2.5_ was related to an 11% elevated risk of chronic kidney disease stage ≥3 at 2 years after donor nephrectomy. Moon et al. [87] concluded that exposure to PM_2.5_ negatively affected renal function and was positively associated with the prevalence of chronic kidney disease in this population. It was also shown that current smokers presented significantly lower eGFR and elevated serum IL-6 levels relative to both past smokers and non-smokers after kidney transplantation [88].

Khalil et al. [89] also discussed systematically the impacts of cigarette smoking in native kidneys, living kidney donors, and kidney transplant recipients. However, according to Abramowicz et al. [90] the prerequisite of non-smoking remains a controversial issue for potential kidney transplant recipients.

The above-mentioned studies focused on the influence of air pollution on native kidneys, but kidney transplant patients are likely to be even more susceptible to detrimental effects of air pollutants. There are several reasons for this hypothesis. Usually, transplant recipients have a greater burden of comorbidities [91,92]. Chronic immunosuppression leads to disturbances in their immune system [92,93,94]. PM oxidative stress may lead to delayed graft function [95,96] and systemic inflammatory response is a risk factor of delayed graft function and graft rejection [97,98,99,100,101].

In addition, limitations of the cited studies include potential confounding bias such as different designs, methodological limitations (different methods of obtaining data on air pollution, different populations transplanted in different time frames), or heterogeneity between studies (no data on whether patients changed their residency, no data on other comorbidities, etc.).

Rasking et al. [19] studied kidney biopsies from 25 transplant patients and focused on the visualization of black carbon particles using white light generation under femtosecond-pulsed illumination. Black carbon particles are ultrafine particles, which can reach the systemic circulation and therefore may distribute to distant organs upon inhalation. They accumulate near different structural components of the kidney and represent a potential mechanism explaining the detrimental effects of particle air pollution exposure on kidney function. Rasking et al. [102] also assessed urinary kidney injury molecule-1 (KIM-1) and cystatin C and found that an increase in these biomarkers in urine of kidney allograft recipients may reflect air-pollution-induced kidney injury. This is a first step in addressing the adverse effects black carbon particles might exert on kidney function. Their study was the first to assess naturally present black carbon particles from air pollution in kidney tissue. They also stressed that the small sample size could be a limitation. Moreover, kidney transplant recipients could also be particularly prone to environmental toxicants, such as black carbon and/or PM_2.5_. In addition, grafts could also be exposed to black carbon through the donor prior to transplantation. Therefore, a healthy lifestyle of the donor may influence recipient graft function. It might also at least in part explain why kidneys from living donors perform far better than those of a deceased donor.

On the other hand, Rasking et al. [103] continued their research on the animal model of wild-type C57BL/6J mice exposed to either HEPA-filtered air or clean ultrafine carbonaceous particles (UFP^C^, 450 µg/m^3^) during the prenatal and/or postnatal phase. They found that, in mice exposed both pre- and postnatally to UPF, cortical and medullary areas as well as tubular and interstitial structures were altered the most while glomeruli and vessels the least. They concluded that these changes could potentially increase kidney vulnerability to injury.

## 4. Clinical Importance of the Relationship Between Air Pollution and Higher Rates of Kidney Disease and KRT

Across three meta-analyses [25,26,27], the direction of effect of air pollution on CKD risk is consistent (higher exposure → higher CKD risk), though effect sizes and pollutant coverage differ. It appears that, taking into consideration these three meta-analyses, these are associations rather than definitive causal estimates; however, dose–response patterns and robustness in sensitivity analyses lend credibility to the observed relationships. Concordant effect sizes are observed in a large European cohort [28] and in the UK Biobank [29].

The three meta-analyses summarize per-increment risk and do not propose thresholds. Two of the studies cited above [28,29] explicitly modeled exposure–response with cubic splines (smooth function) and did not identify a clear threshold.

Overall, current evidence indicates no universal exposure threshold for renal risk from air pollution; hence, the public health message is that lower exposure is better. Consistent with the “no safe level” principle—particularly for PM_2.5_—adverse health effects are observed even at low concentrations, so exposures should be driven as low as reasonably achievable. Reflecting this evidence, the WHO tightened its Global Air Quality Guidelines in 2021 to PM_2.5_: 5 µg/m^3^ (annual) and 15 µg/m^3^ (24 h, 99th percentile ≈ 3–4 exceedances/year); NO_2_: 10 µg/m^3^ (annual) and 25 µg/m^3^ (24-h, 99th percentile) [24].

There is no safe threshold for air-pollution exposure; consistent with the exposure–response literature for PM_2.5_ and NO_2_, renal risk increases even at low concentrations, so levels should be driven as low as reasonably achievable. Globally, ~94% of people are exposed above WHO-recommended levels. In Europe, urban exposure remains pervasive: ~96% of city residents exceed the WHO annual guideline for PM_2.5_ and ~88% exceed the WHO guideline for NO_2_. The highest-risk European hotspots—judged by the annual number of days exceeding the WHO daily PM_2.5_ guideline—are concentrated in the Balkans and Southeastern Europe (e.g., Serbia, North Macedonia, Albania, Montenegro, Bulgaria, Romania), parts of Central/Eastern Europe (notably Poland, Hungary, Slovakia, Czechia), and Mediterranean basins (northern Italy/Po Valley, Greece, Cyprus), often with >200 days/year above the daily WHO limit. These patterns reflect different pollution regimes: winter “classic/Polish” smog from solid-fuel heating, photochemical smog in sunny, stagnant basins, and episodic Saharan dust intrusions that amplify fine-particle loads. Because CKD/ESKD risk tracks long-term PM_2.5_ and NO_2_, these regions should be considered priority targets for mitigation. In short, the absence of a renal safety threshold plus the very large share of the population above WHO guidelines argues for immediate action to reduce exposure. Building on our published analyses in areas of air pollution but focusing on other outcomes, we report estimates stratified by area-level socioeconomic context. In the EP-PARTICLES atrial fibrillation study and the EP-PARTICLES ischemic stroke study the per-increment effects of PM_2.5_ and NO_2_ were consistently larger in the most deprived areas than in the least deprived, with clear graded (quintile) patterns and significance [2,39]. These findings align with the Lancet Regional Health—Europe analysis of acute coronary syndromes, which likewise showed stronger pollution–event associations in populations and regions with markers of social and environmental vulnerability [11]. Although the literature lacks studies directly reporting the impact of socioeconomic status (SES), in our view these findings can be extrapolated from other research and applied to kidney disease epidemiology as well.

Contemporary cohort studies were explicitly designed to mitigate the concern of reverse causation in observational studies. In the two large European cohorts (RoLS and VHM&PP), exposures were assigned prior to outcome and Cox models adjusted for individual- and neighborhood-level socioeconomic covariates, reducing the risk that the findings are driven by sicker patients preferentially residing in more polluted areas [28]. Similar safeguards are present in the UK Biobank prospective analysis, which used restricted cubic splines and broad confounder adjustment while relating long-term PM_2.5_/NO_2_/NO_x_ exposure to incident ESKD [29]. Of course, this remains a limitation of existing analyses, and we will state it explicitly and outline directions for future investigators. Beyond early CKD, multiple studies report associations with kidney failure requiring replacement therapy (dialysis or transplantation). Last, but not least, we do underline that the associative data described and discussed in this review may not have a cause–effect relationship and this is a meaningful limitation of the published studies.

## 5. Clinical and Public Health Action

Unfortunately, there are still few randomized controlled trials directly demonstrating reductions in “hard” kidney endpoints following decreases in air pollution exposure. However, there is substantial indirect evidence: interventional randomized controlled trials (RCTs) using home HEPA air purifiers consistently lower systolic blood pressure by approximately 3 mmHg [104,105,106]; moreover, numerous cohort studies show that improvements in air quality (reductions in PM_2.5_) are associated with a lower risk of CKD development and less frequent initiation of dialysis among individuals with advanced CKD [107,108,109,110,111,112,113]. Taken together, these findings provide a credible basis for action now, even in the absence of large RCTs with renal endpoints. Among the interventions with the strongest evidence are household environmental measures. In our view, these findings underscore the need to develop environmental nephrology and to systematically inform both patients and clinicians about the impact of environmental exposures on kidney health. The key priorities at present are: (1) implementation of practical exposure-reduction strategies, and (2) further research—including RCTs with renal endpoints as well as the application of modern causal-inference methods (e.g., causal DTs)—to strengthen causal evidence and evaluate the impact of interventions on earlier surrogate endpoints such as albuminuria or the rate of eGFR decline. We need coordinated action—from simple household interventions to urban policies—to meaningfully reduce patient exposure. It is crucial to address not only air pollution exposure but all environmental determinants, e.g., noise, soil, wastewater, simultaneously rather than in isolation. At the population level, these include urban and transport reforms to limit traffic in city centers and reduce congestion, the promotion of low- and zero-emission vehicles, the installation of noise barriers in densely populated and sensitive areas, the enforcement of speed limits and the use of silent road surfaces, as well as night-time bans on air traffic and accelerated adoption of quieter engine technologies. The importance of energy transition measures such as shifting to renewable and clean energy sources and developing transport infrastructure to reduce congestion and vehicular pollution is also highlighted. At the individual level, people can reduce exposure using home air purifiers, keeping car and home windows closed during high-pollution episodes, using air-conditioning filters, wearing earplugs or noise-canceling headphones, reducing household noise, avoiding prolonged exposure to loud environments, closing shutters at night, and activating night modes on electronic devices. Finally, the need to focus on vulnerable populations, including the elderly and individuals with cardiovascular or kidney conditions, and to implement combined measures for those living in high-risk environments is emphasized. Together, these recommendations highlight a multilevel approach—urban planning, policy enforcement, energy transition, and individual protection—that is consistent with best practice in environmental epidemiology and public health.

## 6. Conclusions

In recent years, an expanding body of evidence has shown that air pollution adversely affects kidney health, accelerating progression to ESKD and the need for KRT i.e., dialysis, or transplantation. Kidney transplant recipients appear particularly vulnerable. Further in-depth studies are warranted to confirm these associations, elucidate underlying mechanisms, and strengthen causal inference among patients on dialysis and transplant recipients. There is a particular need for RCTs with hard renal endpoints; given the logistical and ethical complexity of such trials, parallel support from artificial intelligence—for example, through causal digital twin models and in silico RCTs—may help accelerate the generation of credible evidence and optimize the design of prospective studies. The existing data can improve understanding of CKD pathogenesis and progression and inform preventive strategies aimed at delaying or avoiding KRT. Reducing air pollution should therefore be a public health priority, with benefits for both quality of life and health economics.

## Figures and Tables

**Table 1:** Effects of air pollution on the development and progression of chronic kidney disease (CKD) to end-stage kidney disease (ESKD).

	Short/Long-Term	Country/Population	Study Design	Pollutants	Health Effects
1. Lou et al. [66]	Short-term	18,114 hemodialysis death cases/China	Case–control study	PM_2.5_ and PM_10_	Each short-term increase of 10 µg/m^3^ in PM_2.5_ and PM_10_ was statistically significantly associated with a relative increase of 1.07% and 0.89% in daily mortality rate of hemodialysis patients.
2. Huh et al. [67]	Short-term	134,478 dialysis patients/Korea	Cohort	CO	A significant association between CO exposure and all-cause mortality.
3. Remigio et al. [68]	Short-term	43,338 ESKD patients/USA	Cohort	PM_2.5_ O_3_	A 10-unit increase in PM_2.5_ concentration was associated with a 5% increase in all-cause mortality and same-day O_3_ after adjusting for extreme heat exposures.
4. Xi et al. [69]	Short-term	314,079 hemodialysis patients/USA	Cohort	PM_2.5_	A 10 µg/m^3^ increase in the average lag 0–1 daily PM_2.5_ exposure was associated with cardiovascular disease (CVD) incidence, CVD mortality, and all-cause mortality.
5. Lin et al. [70]	Long-term	161,970 subjects without CKD/Taiwan	Cohort	PM_2.5_ NO, SO_2_	Compared with Q1-level SO_2_, exposure to the Q4 level was associated with a 1.46-fold higher risk of developing CKD and 1.32-fold higher risk of ESRD. Compared with Q1-level NOx, exposure to the Q4 level was associated with a 1.39-fold higher risk of developing CKD and 1.70-fold higher risk of ESRD. Compared with Q1-level NO, exposure to the Q4 level was associated with a 1.48-fold higher risk of CKD and 1.54-fold higher risk of ESRD. Compared with Q1-level particles <2.5 µm (PM_2.5_), exposure to the Q4 level was associated with a 1.74-fold higher risk of CKD and 1.69-fold higher risk of ESRD.
6. Troost et al. [71]	Long-term	925 patients with primary glomerulopathies/USA	Cohort	PM_2.5_, BC, and sulfate	A doubling of baseline PM_2.5_ and BC was associated with a greater risk of disease progression (ESKD diagnosis or ≥40% reduction in eGFR from baseline). No association with sulfates in unadjusted or adjusted analyses.
7. Wu et al. [72]	Long-term	6480 CKD/ Taiwan	Cohort	PM_2.5_, NO_2_	The risk of eGFR deterioration was found to increase with increasing PM_2.5_ and NO_2_ level especially for those exposed to PM_2.5_ ≥ 31.44 µg/m^3^ or NO_2_ ≥ 15.00 ppb.
8. Blum et al. [73]	Long-term	945,251 dialyzed adults/USA	Retrospective cohort from US Renal Data System	Extreme heat	498,049 adults were exposed to at least 1 of 7154 extreme humid-heat events, 500,025 deaths occurred at the mean follow-up of 3.6 years. Increased risk of death (hazard ratio 1.18 (95% CI, 1.15–1.20)) during extreme humid-heat exposure for dialysis patients, higher relative mortality among patients living in the southeast (*p* < 0.001) compared with the southwest of USA.
9. Xi et al. [74]	Long-term	314,079 hemodialysis patients/USA	Cohort	PM_2.5_	Each 1 µg/m^3^ increase in annual average PM_2.5_ was associated with a greater rate of CV events and CVD-specific mortality.
10. Jung et al. [75]	Long-term	5041 hemodialysis patients/South Korea	Cohort	PM_10_, NO_2_, and SO_2_	A significant long-term relationship between mortality risk and interquartile range (IQR) of increase in PM_10_, NO_2_, and SO_2_.
11. Lin et al. [76]	Long-term	160 peritoneal dialysis (PD) patients	Cohort	NO_2_	Patients undergoing PD and having high environmental NO_2_ exposure have a higher 2-year mortality rate than those with low environmental NO_2_ exposure.

## Data Availability

No new data were generated.
